# Anterior Cingulate epilepsy: mechanisms and modulation

**DOI:** 10.3389/fnint.2013.00104

**Published:** 2014-01-03

**Authors:** Wei-Pang Chang, Bai-Chuang Shyu

**Affiliations:** ^1^Graduate Institute of Life Science, National Defense Medical CenterTaipei, Taiwan; ^2^Institute of Biomedical Science, Academia SinicaTaipei, Taiwan

**Keywords:** cingulate epilepsy, thalamus modulation, epileptogenesis, GABA antagonists, gap junction modulation

## Abstract

Epilepsy is a common neurological disorder, about 1% population worldwide suffered from this disease. In 1989, the International League Against Epilepsy (ILAE) classified anterior cingulate epilepsy as a form of frontal lobe epilepsy (FLE). FLE is the second most common type of epilepsy. Previous clinical studies showed that FLE account an important cause in refractory epilepsy, therefore to find alternative approach to modulate FLE is very important. Basic research using animal models and brain slice have revealed some insights on the epileptogenesis and modulation of seizure in anterior cingulate cortex (ACC). Interneurons play an important role in the synchronization of cingulate epilepsy. Research has shown that the epileptogenesis of seizure originated from mesial frontal lobe might be caused by a selective increase in nicotine-evoked γ-aminobutyric acid (GABA) inhibition, because the application of the GABA_A_ receptor antagonist picrotoxin inhibited epileptic discharges. Gap junctions are also involved in the regulation of cingulate epilepsy. Previous studies have shown that the application of gap junction blockers could attenuate ACC seizures, while gap junction opener could enhance them in an *in vitro* preparation. μ-Opioid receptors have been shown to be involved in the epileptic synchronization mechanism in ACC seizures in a brain slice preparation. Application of the μ-opioid agonist DAMGO significantly abolished the ictal discharges in a 4-aminopyridine induced electrographic seizure model in ACC. Basic research has also found that thalamic modulation has an inhibitory effect on ACC seizures. Studies have shown that the medial thalamus may be a target for deep brain stimulation to cure ACC seizures.

## INTRODUCTION

Seizure is a common neurological disorder that affects approximately 1% of the population worldwide. Frontal lobe epilepsy (FLE) is the second most prevalent type of seizure, but detecting seizure onset in FLE is difficult. Most seizures are detectable because epileptic currents pass through brain areas that are involved in motor or language processing. Limbic seizures are difficult to study because the symptoms in these patients are usually related to alterations in motivational, social, and cognitive function (Csernansky et al., 1990; Levin and Duchowny, 1991). These subtle symptoms are sometimes difficult to detect unless the seizure activity spreads to other brain regions. The ACC is considered a part of the limbic cortex, and the ACC is one of the most difficult brain regions in which to detect seizure onset. This is because the ACC is not readily accessible for routine electrographic investigations using scalp electrodes (Quesney, 1986), and the close proximity between the right and left ACC also increases the difficulty in identifying where seizures actually initiate (Mazars, 1970; Geier et al., 1977; Nadkarni and Devinsky, 2009). Invasive electrodes only provide limited sampling. The dense venous drainage over the medial surface of the hemisphere hinders electrode placement in the ACC. Despite these limitations, clinical studies have provided insights into ACC function, and basic research has revealed the mechanism of epileptic synchronization and how ACC seizures are modulated. Cingulate epilepsy was first characterized and defined in 1970 using an intracerebral-depth electrode (Mazars, 1970) and such seizures are usually classified as simple partial (Nadkarni and Devinsky, 2009).

The ACC can be subdivided into affective and cognitive parts (Bush et al., 2000; Vogt, 2005). The affective part is connected to the periaqueductal gray, amygdala, anterior insula, and nucleus accumbens (Devinsky et al., 1995). The affective pathway is involved in endocrine and autonomic function (Critchley et al., 2005). The cognitive part is interconnected with the parietal cortex, lateral prefrontal cortex, and premotor and supplementary motor areas (Devinsky et al., 1995; Bush et al., 2000). Investigations of seizures can provide additional insights into brain function. The symptoms of ACC seizures are closely associated with interactions between the ACC and other brain regions.

## CLINICAL STUDIES OF CINGULATE EPILEPSY

In the pre-magnetic resonance imaging (MRI) era, clinical studies of ACC seizures were primarily retrospective. The data were obtained from patients with ACC seizures who underwent anterior cingulotomy. Patients who were free from seizures after anterior cingulotomy strongly suggested that the seizure originated in the ACC. With the invention of MRI, magneto encephalogram (MEG), intracranial electrodes, and single-photon emission computed tomography (CT), clinicians were able to more precisely locate the seizure onset in the ACC.

Cingulate epilepsies were first characterized by MRI and CT in 1970 (Mazars, 1970). In 1989, the International League Against Epilepsy included cingulate epilepsy as a type of FLE. The ACC epilepsy was classified as a type of FLE by ILAE in 1989. However, the term “ACC epilepsy” is controversial because the symptoms of ACC epilepsies may overlap with other types of FLE (Williamson et al., 2000). Some researchers have attempted to distinguish cingulate gyrus epilepsy from FLE by examining semiological patterns (Williamson et al., 2000). Other researchers considered that seizures caused by lesions in the cingulate cortex are more specific and can be classified as ACC seizures (Alkawadri et al., 2011).

Patients with ACC seizures fall into two categories: (1) patients with lesions in the ACC, which also includes cortical dysplasia in the ACC that causes focal seizures (Biraben et al., 2001; Nobili et al., 2007), and patients with ACC neoplasms, but this condition is quite rare (Zaatreh et al., 2002); (2) patients with no lesions in the ACC and a normal MRI that reveals only non-specific findings. Most focal ACC epilepsies are believed to be idiopathic and cryptogenic. Clinically, these lesional ACC seizures are often characterized by an early onset, drug resistance, and behavioral disturbances (Biraben et al., 2001; Zaatreh et al., 2002).

Anterior cingulate seizures have a broad range of clinical manifestations. The age of onset of ACC seizures is usually early in life (Williamson et al., 1985). However, ACC seizures may also start in adulthood. ACC seizures mostly occur during sleep and can be misdiagnosed as parasomnias. The common symptoms of ACC seizures include emotional outbursts. Autonomic symptoms are also common (Devinsky et al., 1995; Nadkarni and Devinsky, 2009). In adults, the aggressive features and psychotic symptoms of ACC seizure are overt, but a case report of young children showed intact intellect and normal behavioral ability (De Rose et al., 2009).

These clinical symptoms have been described as seizures that originate in the frontal lobe, and these symptoms are the hallmark of seizures that affect area 24. Despite clinical evidence that demonstrates that the ACC is involved in frontal lobe epileptic disorders, few basic research studies have reported the mechanism of seizure synchronization in the ACC.

## ANIMAL MODELS OF ACC SEIZURES

There are clinical limitations on identifying seizure onset within ACC. Although an invasive depth and subdural electrodes increase spatial resolution in identifying seizure onset, they only provide limited sampling (Quesney, 1986; Quesney et al., 1992). Therefore, animals models are needed to conduct ACC epilepsy research. The first animal model of anterior cingulate seizure was established by (Andy and Chinn, 1957). Threshold and suprathreshold electrical stimulation of the ACC was used to induce epileptic afterdischarges in unanesthetized freely moving cats. The afterdischarges invariably propagated to the contralateral ACC. The propagation between the left and right ACC was faster than between the ACC and posterior cingulate gyrus. The propagation of the cingulate epileptic afterdischarges also passed through various brain structures, such as the sensory cortex basal ganglia, cerebellum, hypothalamus, and mesencephalic structure. Afterdischarges also propagated to the motor cortex but less frequently. Behavioral changes were minimal during cingulate gyrus afterdischarges and only one cat showed extremely aggressive behavior during afterdischarges (Andy and Chinn, 1957).

The kindling (i.e., motor seizure development) model was first established in rodents (Racine, 1975). Kindling in the ACC requires a mean of 11.6 s stimulation. The initial discharges in the frontal-cingulate regions were short in duration, with an average of 10.6 s. The seizures that arose from the ACC showed strong transhemispheric propagation. The electroencephalographic spike of the first afterdischarge was usually simple, with a frequency of 1–3 Hz in the ACC. Approximately 75% of the rodents with seizures that arose from cingulate kindling exhibited an immediate loss of postural control without rearing during the first and subsequent afterdischarges. Approximately 50% of the rodents exhibited these symptoms in the second to fourth afterdischarge. The behavioral seizure response was a mixture of both neocortical and limbic types (Racine, 1975).

Repetitive electrical stimulation of the ACC in baboons (*Papio papio*) also induced cingulate seizures. The symptoms that arose from ACC kindling had protracted non-convulsive seizure state features, such as flexion of the neck, widening of the eyelids, rapid bilateral spread, and eventually secondary generalization. Kindling of the ACC evolves into convulsive seizures after epileptiform activity propagates to the frontal central cortex. Focal epileptogenesis on one side of the ACC was shown to interfere with seizure development on the contralateral side (Wada and Tsuchimochi, 1995). These authors later showed that cingulate kindling can lead to the prolonged inhibition of kindling at a homotopic secondary site. This antiepileptic effect is not specific to primates because it was also shown to occur in cats. They also showed that the antiepileptic effect was not confined to the contralateral homotopic site, and the antiepileptic effect is presumably attributable to an enhanced intrinsic inhibitory mechanism in the mammalian brain (Wada and Hirayasu, 2004).

**FIGURE 1:**
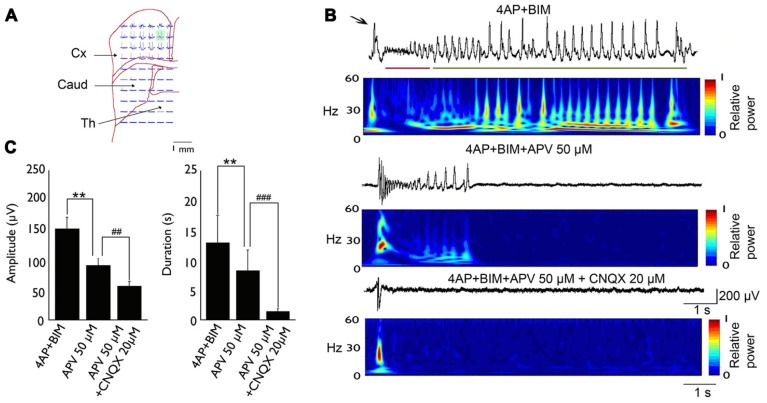
**Typical example of 4-AP + bicuculline-induced epileptiform activity in an MT-ACC slice.** **(A)** Location of the multielectrode and brain slice. The green area is selected and magnified in **(B)**. **(B)** Epileptiform activity is composed of ictal discharges (arrow), tonic-phase firing (red line), and clonic-phase firing (green line). The application of APV (50 μM) decreased the amplitude and duration of clonic-phase firing, and the subsequent application of CNQX (20 μM) completely abolished tonic- and clonic-phase firing. The application of CNQX also decreased the amplitude of ictal discharges. **(C)** The statistical results showed that both the amplitude and duration of epilepsy were significantly decreased after application of APV and CNQX. Comparison were performed using ANOVA and *post hoc* *t*-test (**p < 0.01; ##*p* < 0.01; ###*p* < 0.001). Caud, caudate; Cx, cortex; Th, thalamus. Adapted from Chang et al. (2011).

The basic synchronization mechanism of cingulate epileptiform activity has been studied *in vitro* (Panuccio et al., 2008a, 2009; Chang et al., 2011, 2013). The convulsant 4-aminopyridine (4-AP) induces epileptic discharges in humans (Lundh et al., 1984) and other mammals (Glover, 1982). *In vitro* studies showed that 4-AP could induce epileptic events in different brain regions, such as the cingulate cortex (Panuccio et al., 2009), amygdala (Klueva et al., 2003), and parahippocampal cortex (Avoli et al., 1996). 4-AP is a potassium channel blocker that affects A-type and D-type K^+^ currents (Ulbricht and Wagner, 1976; Storm, 1988). 4-AP-induced seizures are sensitive to anticonvulsants, and pharmacoresistant activity can be induced by combining the GABA_A_ receptor antagonist bicuculline with 4-AP (Bruckner et al., 1999). Bath application of 50 μM 4-AP in coronal ACC slices elicited epileptiform synchronization that was composed of interictal and ictal events. The glutamatergic system is involved in the epileptic synchronization of cingulate epilepsy. In a 4-AP-induced seizure model, bath application of the *N*-methyl-D-aspartate (NMDA) receptor antagonist CPP (10 μM) abolished ictal events but did not affect interictal events. Concomitant application of the AMPA/kainate receptor antagonist CNQX (10 μM) abolished ictal events and reduced the amplitude of interictal events (Panuccio et al., 2009). In a 4-AP + bicuculline-induced drug-resistant seizure model, the application of the NMDA receptor antagonist APV (50 μM) shortened the duration and amplitude of clonic phase discharge. Concomitant application of the AMPA/kainate receptor antagonist CNQX (20 μM) completely suppressed tonic- and clonic-phase seizures (Chang et al., 2011; **Figure 1**).

Application of the GABA_A_ and GABA_B_ receptor antagonists PTX (50 μM) and CGP55845 (4 μM), respectively, abolished ictal events induced by 4-AP and transformed the epileptiform activity into recurrent synchronous discharges. The results showed that GABA_A_ transmission contributed to the synchronization of epileptic discharges in the ACC (Panuccio et al., 2009). The paradoxical GABA-mediated excitatory mechanism may result from a shift of GABA_A_ receptor reversal potential (Staley and Proctor, 1999) or a transient increase in [K^+^]_0_, which in turn enhances synchronization through a synaptic or non-synaptic mechanism (Avoli et al., 1996; Kohling et al., 2000; Gigout et al., 2006). The synchronization of ACC seizures is also mediated by gap junctions (Panuccio et al., 2008a; Chang et al., 2013). The application of a gap junction blocker significantly decreased the amplitude and duration of epileptiform activity. Epileptic synchronization in the ACC is also subjected to opioid modulation. Application of 10 μM DAGO significantly abolished ictal discharges induced by 4-AP (Panuccio et al., 2009).

In cortical areas, the lateral propagation of electrical activity is under tight control because unrestrained, laterally propagated electrical activity easily leads to epileptiform activity. Epileptiform activity in neocortical areas is restrained by surrounding inhibition (Prince, 1967), and a decrease in surrounding inhibition causes the spread of epileptiform activity (Pinto et al., 2005). The development of interneurons in the ACC was altered in mice with targeted mutation of the gene that encodes urokinase plasminogen activator receptor (μPAR). The ACC and parietal cortical areas showed 50% fewer GABAergic interneurons in a *μPAR*^-/-^ mouse strain compared with wildtype littermates. The numbers of interneurons in other cortical areas did not differ from wildtype mice. The *μPAR*^-/-^ strain displayed spontaneous seizures and a lower seizure threshold when challenged with pentylenetetrazol (Powell et al., 2003). Although seizure onset was not determined, the *μPAR*^-/-^ strain may serve as an animal model for investigating the importance of GABAergic interneurons in ACC seizures.

Epileptiform activity that initiates in the ACC showed strong trans-hemispheric propagation. To test whether left and right ACC epileptic discharges synchronize through the corpus callosum, a modified slice cutting method was established to preserve the corpus callosum between the left and right ACC (Walker et al., 2012). Seizure-like activity could be induced by the bath or local application of bicuculline and in a zero-magnesium solution. Seizure-like activity could be regulated by the corpus callosum, demonstrated by an incision of the callosum *in vitro*. An incision of the callosum diminished bilateral propagation. Interestingly, patch-clamp recordings showed that inhibitory postsynaptic currents (IPSCs) were increased by the focal application of bicuculline in the contralateral ACC. No GABAergic projection was found between the left and right ACC, and the authors concluded that the callosal projection has a strong effect on local GABAergic interneurons (Walker et al., 2012).

## EPILEPTOGENESIS MECHANISM OF ACC SEIZURES

Most ACC seizures in clinical cases result from lesions. The remainder of ACC seizures are sporadic, and the pathophysiological mechanisms appear to be similar to those affect the cerebral cortex. Cortical epilepsies can result from extracellular ionic fluctuations (Taylor and Dudek, 1982), the dysfunction of energy metabolism (Cavus et al., 2005), channelopathies (Kullmann, 2002), and alterations in transmitter uptake (Chapman, 1998; Rainesalo et al., 2004). Although the pathophysiological mechanisms may have major differences, the outcome of the hypersynchronous bursting of cortical neurons and the concomitant phenotype are similar if the same brain regions are involved.

Clinical electroencephalographic and functional MRI (fMRI) data suggest that autosomal-dominant frontal lobe epilepsy (ADFLE) may have a mesial frontal origin (So, 1998). ADFLE often involves complex motor movements and vocalizations. The gene loci that encode the nicotinic acetylcholine receptor α and β subunits CHRNA4, CHRNB2, and CHRNA2 are involved in ADFLE (Steinlein et al., 1995; Bertrand et al., 1998; De Fusco et al., 2000). Two mouse strains that carry mutant alleles of the α4 subunit of the nicotinic acetylcholine receptor display spontaneous seizures. *In vitro* recordings of neocortical pyramidal neurons showed that nicotine-evoked GABAergic inhibition is significantly increased. Spontaneous seizures could be blocked by the application of a low dose of the GABA_A_ receptor antagonist picrotoxin. These results suggest that excessive GABAergic transmission is involved in the epileptogenesis of ACC seizures. Epilepsy that occurs in the ACC may be attributable to enhanced GABAergic function (Engel, 1996; Mann and Mody, 2008; Panuccio et al., 2008b). The application of the GABA_A_ receptor antagonist picrotoxin inhibited epileptic discharges (Klaassen et al., 2006). The possible mechanisms of GABAergic inhibition that contribute to epileptogenesis include the resetting of synchronization (Klaassen et al., 2006), the direct excitatory effects of axo-axonic interneurons in layer II/III pyramidal cells (Szabadics et al., 2006), or changes in GABA reversal potential (Marty and Llano, 2005).

The dysregulation of interneuron development might also contribute to abnormal epileptic discharges (Levitt et al., 2004). The *μPAR*^-/-^ mouse strain exhibited a specific reduction of parvalbumin-positive interneurons in the ACC and parietal cortex and displayed spontaneous seizures. Previous studies showed that the hypersynchrony of GABAergic transmission is involved in ACC seizures (Panuccio et al., 2009). These results indicate that the balance between excitatory and inhibitory transmission is very important in seizure control, and the dysregulation of GABAergic transmission is one of the factors of the epileptogenesis of ACC seizures.

## MODULATION OF ACC SEIZURES

### THALAMIC MODULATION OF ACC SEIZURES

Seizure onset in limbic systems might propagate to different limbic sites and some nuclei in the thalamus, such as parafasicular nuclei (Langlois et al., 2010), mediodorsal nucleus (Juhasz et al., 1999), and centromedian nucleus (Velasco et al., 1995). Thalamic nuclei are involved in communication between different cortical regions and also support seizure propagation between a primary focus and other cortical and subcortical regions. Therefore, these nuclei could play a pivotal role in the remote control of seizure activity and be an interesting target for DBS (Kahane and Depaulis, 2010). The ACC is reciprocally connected with the MT (Vogt et al., 1987; Hatanaka et al., 2003; Vogt, 2005), and the MT might play a pivotal role in the remote control of seizure synchronization (Kahane and Depaulis, 2010).

Previous studies demonstrated that the MT is involved in seizure modulation, especially seizures that involve limbic regions. The MT has been consistently shown to be involved in seizure onset. A significant amount of neuronal loss can be found in medial dorsal and rhomboid/reuniens nuclei. These results suggest that the MT plays a role in limbic seizure modulation (Bertram et al., 1998, 2001). Clinical studies showed that electrical stimulation of the MT decreases the occurrence of seizures (Sterman et al., 1982; Urino et al., 2010), and these results indicate that MT activity is involved in seizure blockade.

Studies of the mechanisms of seizure generation have used the genetic Absence Epilepsy Rat from Strasbourg and showed that spike-wave discharges (SWDs) can be generated from within the somatosensory cortex (Polack et al., 2009). When thalamic activity was blocked by tetrodotoxin (TTX), cortical epileptiform activity turned into a longer sequence of SWDs, indicating that thalamic inputs might suppress epileptic activity. The prolongation of epileptiform activity could be attributable to desynchronization following tonic firing in ventral–medial thalamocortical (TC) neurons (Glenn et al., 1982). Thalamic inputs might desynchronize the cortical response. Previous studies showed that noxious stimulation can increase medial thalamic activity and desynchronize the cortical electroencephalogram (Antognini et al., 2000).

**FIGURE 2:**
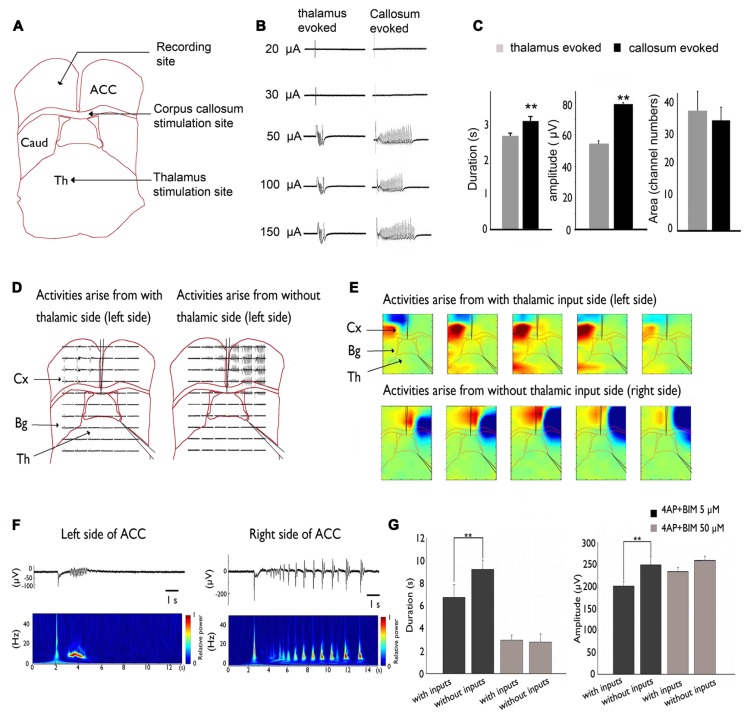
**Inhibitory effect of MT stimulation on 4AP + bicuculline-induced epileptiform activity.** **(A)** Stimulation sites in the MT and CC and recording site in the ACC. **(B)** Activity evoked by MT and CC stimulation. Evoked responses were all-or-none because they were not altered by changes in stimulation intensity. **(C)** The duration and amplitude of epileptiform activity were significantly greater in response to CC stimulation than in response to MT stimulation. **(D)** A cut was made in the middle of the corpus callosum and at the border between the thalamus and basal ganglia. Epileptiform activity that arose from the side without thalamic inputs was significantly larger than the activity that arose from the side with thalamic inputs. **(E)** Pseudocolor isopotential map that shows that the seizure began within the cortex and propagated to the basal ganglia and thalamus. **(F)** Typical traces were selected and magnified. Notice that the duration and amplitude of epileptiform activity were larger on the right side of the ACC (i.e., thalamic input removal side). **(G)** Summary results that show that the amplitude and duration of epileptiform activity were significantly larger in the thalamic removal groups. However, when the concentration was increased to 50 μM to completely block GABAergic transmission, no significant difference was observed between groups. Comparison were performed using ANOVA and *post hoc* *t*-test (**p < 0.01). Caud, caudate; Cx, cortex; Th, thalamus. Adapted from Chang et al. (2011).

Our recent studies used brain slices that preserved the pathway between the MT and ACC (Lee et al., 2007). We showed that thalamic inputs could desynchronize epileptic events in the 4-AP + bicuculline-induced seizure model (Chang et al., 2011 and **Figure 2**).

This inhibitory effect on seizure activity might occur via the activation of GABAergic transmission. Our results showed that 50 μM bicuculline completely suppressed the GABAergic system, and no significant difference was found between the intact TC and severed thalamic groups. Thus, thalamic inputs may exert inhibitory effects via the GABAergic system in the ACC.

The lateral propagation of seizure-like activity in the neocortex is restrained by surrounding inhibition. Studies of hippocampal slices also showed that epileptiform activity became synchronized in different columns when surrounding inhibition collapsed. Thalamic inputs might activate and strengthen surrounding inhibition. This phenomenon was demonstrated in a calcium imaging experiment, in which calcium transients tended to be more synchronized when the inhibitory effect of thalamic inputs was eliminated. Removing the thalamic inputs in our slice may have decreased the inputs that activate surrounding inhibition or desynchronized them, causing epileptiform activity to wane (Chang et al., 2011).

### μ-OPIOID RECEPTOR AGONIST MODULATES ACC SEIZURES

The opioid receptor family includes the μ, δ, and κ receptors (Benarroch, 2012). The opioid receptors are expressed throughout the central nervous system. The high expression of opioid ligand binding sites can be found in the limbic system and ACC, the major region of opioid action in the brain (Herz et al., 1970; Hiller et al., 1973; Pert and Yaksh, 1974). All three opioid receptor subtypes are localized in the ACC, but the relative amount of κ receptors is less, and their distribution varies among different layers (Mansour et al., 1987). Opioid receptors in the ACC are known to be involved in the top-down modulation of pain signals (Petrovic et al., 2002; Eippert et al., 2009), the incentive motivational properties of drug-related cues (Gremel et al., 2011), and affective responses (Zubieta et al., 2003).

Endogenous opioids in the brain could act as neurohormonal transmitters for epilepsy (Loacker et al., 2007; Kauffman et al., 2008). Clinical research showed that β-endorphin levels are correlated with seizure frequency and duration (Marek et al., 2010). A positron emission tomography radioligandbinding assay showed that opioid receptor availability was upregulated after spontaneous seizures (Hammers et al., 2007). These clinical studies showed that opioids play an important role in seizure modulation. Previous studies showed that an increase in the level of endogenous opioids increases seizure threshold (Stogmann et al., 2002). The κ receptor agonist dynorphin is released during focal hippocampal seizures to prevent secondary generalization and status epilepticus (Koepp et al., 1998; Romualdi et al., 1999). However, other reports indicated that opioid receptors have biphasic effects with regard to epileptogenesis. At low concentrations, morphine has antiseizure effects, whereas higher concentrations enhanced spontaneous seizures. The proseizure effect of high-dose morphine is mediated through μ and κ receptors, and δ receptor activation appears to not be involved in this process (Saboory et al., 2007).

The role of μ-opioid receptors in the regulation of ACC seizures has been investigated. μ-Opioid receptors have been shown to be involved in the epileptic synchronization mechanism of ACC seizures in brain slice preparations (Panuccio et al., 2009). The bath application of 10 μM [D-Ala^2^, *N*-MePhe^4^, Gly-ol]-enkephalin (DAMGO) significantly abolished ictal discharges induced by 4-AP. This effect could be reversed by the application of 10 μM naloxone (Panuccio et al., 2009).

DAMGO might act on μ-opioid receptors on interneurons to interfere with seizures caused by synchronization of the GABAergic system. The application of DAMGO significantly increased both the duration and interval of the occurrence of epileptic events when the GABAergic system is further blocked by the application of the GABA_A_ and GABA_B_ receptor antagonists PTX (50 μM) and CGP55845 (4 μM), respectively. Alterations in epileptic events induced by DAMGO is thought to occur via interactions with glutamatergic receptors (Panuccio et al., 2009). Thus, both excitatory and inhibitory epileptic synchronization mechanisms in the ACC appear to be modulated by μ receptors.

### GAP JUNCTION MODULATION OF ACC SEIZURES

Gap junctions mainly exist between interneurons in the neocortex (Galarreta and Hestrin, 1999) and are important in the regulation of synchronization between interneurons. Therefore, gap junctions in the ACC might be involved in pathophysiological hypersynchronization in epileptic discharges. Gap junctions are also expressed on glial cells (Nemani and Binder, 2005). Glial cells regulate the ionic concentration in the extracellular space during seizures, preventing the accumulation of potassium that causes neurons to become more excitable (Park and Durand, 2006). Glial cells also regulate the potassium concentrations after seizure activity (Xiong and Stringer, 1999). Gap junctions might be involved in epileptogenesis, especially in the modulation of the spatiotemporal properties and changes in frequency distribution.

Gap junctions are involved in oscillations with different frequencies. These oscillations include theta oscillations (Konopacki et al., 2004; Allen et al., 2011), gamma oscillations (Tamas et al., 2000; Hormuzdi et al., 2001), and fast ripples (Grenier et al., 2003). Previous studies showed that gap junction blockers could block carbachol-induced theta oscillations in brain slices (Konopacki et al., 2004), whereas the gap junction opener TriMA increased theta oscillations (Bocian et al., 2011). This was caused by the local synchronization and desynchronization of interneurons. Using an MT-ACC slice preparation, we found that theta oscillations significantly decreased after application of the gap junction decoupler CBX, indicating that the activity of local interneurons was desynchronized (Chang et al., 2013). Interneurons are important in the synchronization (Engel, 1996; Mann and Mody, 2008; Panuccio et al., 2008b) and restraint of the propagation of seizure-like activity (Prince, 1967; Pinto et al., 2005). The prevalence of gap junctions in cortical interneurons suggests that gap junctions play important roles in seizure propagation. The gap junction decoupler CBX could slow down and desynchronize spontaneous field events. The epileptic discharges were abolished by CBX, and this effect partially recovered with washout (Panuccio et al., 2008a).

**FIGURE 3:**
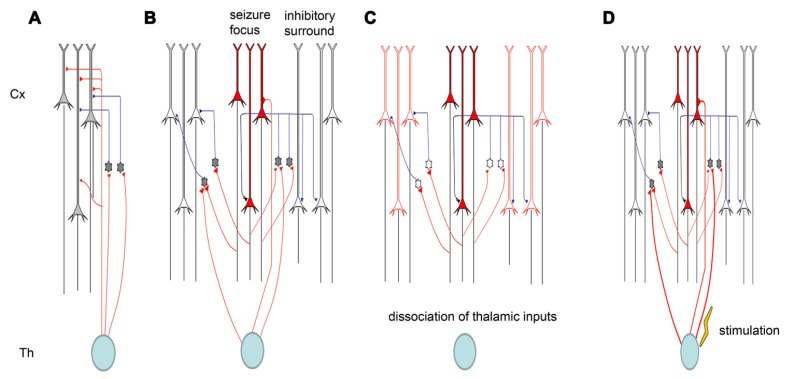
**Schematic diagram of thalamic modulation of cingulate seizures. (A)** The thalamus innervates both pyramidal neurons and inhibitory interneurons in the cortical area. Thalamic inputs can activate inhibitory interneurons, and the interneurons can in turn silence cortical pyramidal neurons. **(B)** Epileptiform activity is usually caused by the hyperactivity of some pyramidal neurons. The activation of these pyramidal neurons will also activate the inhibitory interneurons around them, forming surrounding inhibition that can prevent the lateral propagation of epileptiform activity. **(C)** Removing the thalamic inputs also reduces the inputs of inhibitory interneurons, and the weakening of surrounding inhibition facilitates the propagation of epileptiform activity. **(D)** Electrical stimulation of the thalamus will activate both pyramidal neurons and inhibitory interneurons in cortical areas, but the synapses between thalamic relay neurons and interneurons are stronger than those between thalamic relay neurons and pyramidal neurons. Therefore, electrical stimulation of the thalamus can suppress seizure propagation.

Electrical synapses in the TC system are strong. When electrically coupled cells in the neocortex are excited by thalamic inputs, they typically display strong synchrony of both subthreshold voltage fluctuations and spikes (Cruikshank et al., 2005). The ACC is heavily connected with the MT (Hatanaka et al., 2003; Wang and Shyu, 2004). Our recent studies showed that inputs from the MT could modulate seizure-like activity in the ACC (Chang et al., 2011). The modulation occurs partially through the regulation of cortical gap junctions. One of the important features of TC afferents is that they contact both excitatory projection neurons and local inhibitory interneurons in the cortex. Thus, somatosensory information is immediately distributed to both excitatory and inhibitory cells. Surprisingly, however, the synapses between thalamic relay neurons and inhibitory interneurons are much stronger than those between thalamic relay neurons and excitatory principal cells. Thus, TC afferents lay the foundation for a powerful and simple disynaptic circuit that provides feed-forward inhibition. We found that the removal of thalamic inputs could potentiate cingulate seizure-like activity (Chang et al., 2011), indicating that thalamic inputs exert their effects through cortical interneurons. We also found that electrical stimulation in the thalamus could suppress seizures, and this might also be caused by the activation of cortical interneurons (**Figure 3**).

Gap junctions are significantly involved in the regulation of the clonic phase of seizure-like activity in the cingulate cortex. In our study, we found that ictal bursts and the tonic phase of seizure-like activity, clinically manifested as the tonic phase of a generalized seizure (Logan et al., 2011), are not influenced by a gap junction opener or blocker, while clonic phase is enhanced by the application of a gap junction opener and inhibited by a gap junction blocker. This is because the synchronization and propagating mechanism of ictal bursts and the tonic phase of seizure-like activity induced by 4-AP and bicuculline depend on synaptic transmission mediated by both AMPA and NMDA receptors (Perreault and Avoli, 1992; Borck and Jefferys, 1999; Kohling et al., 2001), and gap junctions are not involved in synaptically synchronized primary bursting activity (Kohling et al., 2001). These results indicate that gap junctions are more involved in the maintenance and propagation of seizure-like activity.

The involvement of gap junctions in the maintenance of seizure-like activity was also demonstrated by the application of a gap junction blocker 30 min prior to the application of 4-AP and bicuculline. Our results showed that the application of a gap junction blocker did not influence the induction of seizure-like activity. 4-AP- and bicuculline-induced seizure-like activity reached a maximal response 50 min after application. Within 50 min, the amplitude and duration of seizure-like activity were not significantly different between the CBX and 4-AP + bicuculline groups, indicating that gap junctions are not involved in the induction stage of seizure-like activity. The significant decrease in the duration of seizure-like activity by the action of CBX is likely mediated by depression of the synchronization between neurons (Szente et al., 2002). Although CBX is also a mineralocorticoid agonist, such receptors are not involved in seizure-like activity induced by 4-AP or a Mg^2+^-free solution (Ross et al., 2000). The results of application of the mineralocorticoid antagonist SPL excluded the possibility that CBX might also act on this receptor.

### CURRENT STIMULATION MODULATION OF FRONTAL LOBE EPILEPSY

Thirty percent of seizure patients suffer from drug-resistant seizures (Kwan and Brodie, 2000). An alternative method has been adopted in clinical research to control seizures. These methods include transcranial magnetic stimulation (TMS), transcranial direct current stimulation (tDCS), and DBS. One of the clinical methods used to cure these patients is DBS. Deep brain stimulation was adopted because it could cure patients with unidentifiable seizure initiation sites, or it could be used to treat patients with a seizure focus that cannot be removed. One of the targeted brain regions for DBS is the thalamus. The thalamus relay information from peripheral to central locations and is responsible for the synchronization of different cortices. Therefore, some nuclei in the thalamus, such as the centromedian, mediodorsal, and parafasicular nuclei, are potential clinical targets for DBS (Bertram et al., 2001; Kahane and Depaulis, 2010). Previous clinical studies showed that anterior thalamus stimulation (4–5 V, 90–110 Hz, 60–90 μV) could alleviate intractable cingulate seizures (Lim et al., 2007). The possible underlying mechanism could be that DBS in the thalamus changes cortical synaptic plasticity (Anderson et al., 2004, 2006). TMS and tDCS are non-invasive methods used to transiently alter neuronal excitability. Transcranial direct current stimulation can transiently alter neuronal excitability, and it is economical compared with TMS. Therefore, many research laboratories and even computer game companies use TDS to influence the subject’s attention or learning and memory ability. The effect of tDCS can outlast the stimulation period (Nitsche et al., 2007) and alter synaptic plasticity (Fritsch et al., 2010). One of the hallmarks of epileptic seizures is enhanced neuronal excitability, and tDCS has been shown to reduce seizures by the diminution of cortical excitability (Nitsche and Paulus, 2009). Transcranial direct current stimulation is particularly useful in patients with epileptogenic foci in cortical convexity (Nitsche and Paulus, 2009). The tDCS stimulation protocol has two modalities. In the closed-loop modality, tDCS is delivered after the epileptic discharge is detected online. In the open-loop modality, a predetermined pattern of stimulation frequency is delivered, regardless of the underlying cortical oscillation. Transcranial direct current stimulation was shown to suppress seizures when applied during interictal states or terminate frontal lobe epileptiform discharges (Kimiskidis et al., 2013). The tDCS of the epileptogenic zone has the potential to control intractable seizures (Morrell, 2006). The nature of the tDCS-induced effect depends on the stimulation parameters, such as the duration, frequency, intensity, and field orientation. Transcranial direct current stimulation is known to cause changes in synaptic plasticity (Fritsch et al., 2010). Transcranial direct current stimulation may suppress seizures long-term by inducing long-term depression.

## CONCLUSION AND FUTURE PERSPECTIVE

The synchronization mechanism of ACC epileptic discharges is largely attributable to the dysregulation of interneuronal networks. Spontaneous seizures are caused by excessive GABAergic transmission, such as in the case of the ADFLE and 4-AP-induced epilepsy models. The reduction of GABAergic transmission in the ACC might also cause seizures, such as the spontaneous seizures found in the *μPAR*^-/-^ mouse strain. To fully understand the role of inhibitory interneurons in ACC seizures, one must precisely control the activity of interneuronal networks by either enhancing or suppressing interneurons. Based on the basic research, the ACC seizure could be modulated by gap junction. Application of gap junction uncoupler decrease the duration of seizure-like activities, while gap coupler enhance seizures. The μ-opioid receptors are also involved in the pathogenesis of ACC seizure, as μ-opioid agonist DAMGO reduced the ictal discharge. The ACC seizure is also subjected to the modulation by thalamic inputs. Removing or inactivating thalamus enhanced seizure-like activities in ACC.

To fully understand the role of inhibitory interneurons in ACC seizures, one must precisely control the activity of interneuronal networks by either enhancing or suppressing interneurons. Optogenetics is the integration of optics and genetics to allow the expression of light-sensitive channels, such as channel rhodopsin-2 (ChR2) and Halorhodopsin from *Natronomonas* (NpHR) in certain groups of neurons (Nagel et al., 2003; Boyden et al., 2005; Zhang et al., 2006). Using blue light to activate ChR2 can trigger action potentials in neurons. Using yellow light to activate NpHR can hyperpolarize neurons. Many knock-in mouse lines are available to specifically and robustly increase the Cre-dependent expression of ChR2 and other light-sensitive proteins (Madisen et al., 2012). By combining hundreds of available Cre lines, ChR2 or NpHR can be expressed in different subtypes of interneurons, and these knock-in mice will facilitate investigations of the function of neural circuits with high fidelity and accuracy.

## Conflict of Interest Statement

The authors declare that the research was conducted in the absence of any commercial or financial relationships that could be construed as a potential conflict of interest.

## References

[B1] Alkawadri R. Mickey B. E. Madden C. J Van Ness P. C. (2011). Cingulate gyrus epilepsy: clinical and behavioral aspects, with surgical outcomes. *Arch. Neurol.* 68 381–385 10.1001/archneurol.2011.21 21403025 PMC5123734

[B2] Allen K. Fuchs E. C. Jaschonek H. Bannerman D. M. Monyer H. (2011). Gap junctions between interneurons are required for normal spatial coding in the hippocampus and short-term spatial memory. *J. Neurosci.* 31 6542–6552 10.1523/JNEUROSCI.6512-10.2011 21525295 PMC3160467

[B3] Anderson T. Hu B. Pittman Q. Kiss Z. H. (2004). Mechanisms of deep brain stimulation: an intracellular study in rat thalamus. *J. Physiol.* 559 301–313 10.1113/jphysiol.2004.064998 15218068 PMC1665080

[B4] Anderson T. R. Hu B. Iremonger K. Kiss Z. H. (2006). Selective attenuation of afferent synaptic transmission as a mechanism of thalamic deep brain stimulation-induced tremor arrest. *J. Neurosci.* 26 841–850 10.1523/JNEUROSCI.3523-05.2006 16421304 PMC6675364

[B5] Andy O. J. Chinn R. M. (1957). Cingulate gyrus seizures; correlation of electroencephalographic and behavioral activity in the cat. *Neurology* 7 56–68 10.1212/WNL.7.1.56 13400211

[B6] Antognini J. F. Carstens E. Sudo M. Sudo S. (2000). Isoflurane depresses electroencephalographic and medial thalamic responses to noxious stimulation via an indirect spinal action. *Anesth. Analg.* 91 1282–1288 11049923

[B7] Avoli M. Barbarosie M. Lucke A. Nagao T. Lopantsev V. Kohling R. (1996). Synchronous GABA-mediated potentials and epileptiform discharges in the rat limbic system in vitro. *J. Neurosci.* 16 3912–3924 8656285 10.1523/JNEUROSCI.16-12-03912.1996 PMC6578615

[B8] Benarroch E. E. (2012). Endogenous opioid systems: current concepts and clinical correlations. *Neurology* 79 807–814 10.1212/WNL.0b013e3182662098 22915176

[B9] Bertram E. H. Mangan P. S. Zhang D. Scott C. A. Williamson J. M. (2001). The midline thalamus: alterations and a potential role in limbic epilepsy. *Epilepsia* 42 967–978 10.1046/j.1528-1157.2001.042008967.x 11554881

[B10] Bertram E. H. Zhang D. X. Mangan P. Fountain N. Rempe D. (1998). Functional anatomy of limbic epilepsy: a proposal for central synchronization of a diffusely hyperexcitable network. *Epilepsy Res.* 32 194–205 10.1016/S0920-1211(98)00051-5 9761320

[B11] Bertrand S. Weiland S. Berkovic S. F. Steinlein O. K. Bertrand D. (1998). Properties of neuronal nicotinic acetylcholine receptor mutants from humans suffering from autosomal dominant nocturnal frontal lobe epilepsy. *Br. J. Pharmacol.* 125 751–760 10.1038/sj.bjp.0702154 9831911 PMC1571006

[B12] Biraben A. Taussig D. Thomas P. Even C. Vignal J. P. Scarabin J. M. (2001). Fear as the main feature of epileptic seizures. *J. Neurol. Neurosurg. Psychiatry* 70 186–191 10.1136/jnnp.70.2.186 11160466 PMC1737203

[B13] Bocian R. Posluszny A. Kowalczyk T. Kazmierska P. Konopacki J. (2011). Gap junction modulation of hippocampal formation theta and local cell discharges in anesthetized rats. *Eur. J. Neurosci.* 33 471–481 10.1111/j.1460-9568.2010.07545.x 21226774

[B14] Borck C. Jefferys J. G. (1999). Seizure-like events in disinhibited ventral slices of adult rat hippocampus. *J. Neurophysiol.* 82 2130–2142 10561393 10.1152/jn.1999.82.5.2130

[B15] Boyden E. S. Zhang F. Bamberg E. Nagel G. Deisseroth K. (2005). Millisecond-timescale, genetically targeted optical control of neural activity. *Nat. Neurosci.* 8 1263–1268 10.1038/nn1525 16116447

[B16] Bruckner C. Stenkamp K. Meierkord H. Heinemann U. (1999). Epileptiform discharges induced by combined application of bicuculline and 4-aminopyridine are resistant to standard anticonvulsants in slices of rats. *Neurosci. Lett.* 268 163–165 10.1016/S0304-3940(99)00341-9 10406030

[B17] Bush G. Luu P. Posner M. I. (2000). Cognitive and emotional influences in anterior cingulate cortex. *Trends Cogn. Sci.* 4 215–222 10.1016/S1364-6613(00)01483-2 10827444

[B18] Cavus I. Kasoff W. S. Cassaday M. P. Jacob R. Gueorguieva R. Sherwin R. S. (2005). Extracellular metabolites in the cortex and hippocampus of epileptic patients. *Ann. Neurol.* 57 226–235 10.1002/ana.20380 15668975

[B19] Chang W. P. Wu J. J. Shyu B. C. (2013). Thalamic modulation of cingulate seizure activity via the regulation of gap junctions in mice thalamocingulate slice. *PLoS ONE* 8:e62952 10.1371/journal.pone.0062952 PMC3653920 23690968

[B20] Chang W. P. Wu J. S. Lee C. M. Vogt B. A. Shyu B. C. (2011). Spatiotemporal organization and thalamic modulation of seizures in the mouse medial thalamic-anterior cingulate slice. *Epilepsia* 52 2344–2355 10.1111/j.1528-1167.2011.03312.x 22092196

[B21] Chapman A. G. (1998). Glutamate receptors in epilepsy. *Prog. Brain Res.* 116 371–383 10.1016/S0079-6123(08)60449-5 9932389

[B22] Critchley H. D. Tang J. Glaser D. Butterworth B. Dolan R. J. (2005). Anterior cingulate activity during error and autonomic response. *Neuroimage* 27 885–895 10.1016/j.neuroimage.2005.05.047 15996878

[B23] Cruikshank S. J. Landisman C. E. Mancilla J. G. Connors B. W. (2005). Connexon connexions in the thalamocortical system. *Prog. Brain Res.* 149 41–57 10.1016/S0079-6123(05)49004-4 16226575

[B24] Csernansky J. G. Leiderman D. B. Mandabach M. Moses J. A. Jr (1990). Psychopathology and limbic epilepsy: relationship to seizure variables and neuropsychological function. *Epilepsia* 31 275–280 10.1111/j.1528-1157.1990.tb05376.x 2344845

[B25] De Fusco M. Becchetti A. Patrignani A. Annesi G. Gambardella A. Quattrone A. (2000). The nicotinic receptor beta 2 subunit is mutant in nocturnal frontal lobe epilepsy. *Nat. Genet.* 26 275–276 10.1038/81566 11062464

[B26] De Rose M. Luzi M. Trignani R. Passamonti C. Zamponi N. Lavano A. (2009). Cingulate epilepsy in a child with a low-grade glioma. *Childs Nerv. Syst.* 25 1507–1511 10.1007/s00381-009-0919-2 19506888

[B27] Devinsky O. Morrell M. J. Vogt B. A. (1995). Contributions of anterior cingulate cortex to behaviour. *Brain* 118(Pt 1) 279–306 10.1093/brain/118.1.279 7895011

[B28] Eippert F. Bingel U. Schoell E. D. Yacubian J. Klinger R. Lorenz J. (2009). Activation of the opioidergic descending pain control system underlies placebo analgesia. *Neuron* 63 533–543 10.1016/j.neuron.2009.07.014 19709634

[B29] Engel J. Jr (1996). Excitation and inhibition in epilepsy. *Can. J. Neurol. Sci.* 23 167–174 8862837 10.1017/s0317167100038464

[B30] Fritsch B. Reis J. Martinowich K. Schambra H. M. Ji Y. Cohen L. G. (2010). Direct current stimulation promotes BDNF-dependent synaptic plasticity: potential implications for motor learning. *Neuron* 66 198–204 10.1016/j.neuron.2010.03.035 20434997 PMC2864780

[B31] Galarreta M. Hestrin S. (1999). A network of fast-spiking cells in the neocortex connected by electrical synapses. *Nature* 402 72–75 10.1038/47029 10573418

[B32] Geier S. Bancaud J. Talairach J. Bonis A. Szikla G. Enjelvin M. (1977). The seizures of frontal lobe epilepsy. A study of clinical manifestations. * Neurology* 27 951–958 10.1212/WNL.27.10.951 561909

[B33] Gigout S. Louvel J. Kawasaki H. D’antuono M. Armand V. Kurcewicz I. (2006). Effects of gap junction blockers on human neocortical synchronization. *Neurobiol. Dis.* 22 496–508 10.1016/j.nbd.2005.12.011 16478664

[B34] Glenn L. L. Hada J. Roy J. P. Deschenes M. Steriade M. (1982). Anterograde tracer and field potential analysis of the neocortical layer I projection from nucleus ventralis medialis of the thalamus in cat. *Neuroscience* 7 1861–1877 10.1016/0306-4522(82)90003-3 6290938

[B35] Glover W. E. (1982). The aminopyridines. *Gen. Pharmacol.* 13 259–285 10.1016/0306-3623(82)90046-5 6127278

[B36] Gremel C. M. Young E. A. Cunningham C. L. (2011). Blockade of opioid receptors in anterior cingulate cortex disrupts ethanol-seeking behavior in mice. *Behav. Brain Res.* 219 358–362 10.1016/j.bbr.2010.12.033 21219940 PMC3062680

[B37] Grenier F. Timofeev I. Steriade M. (2003). Neocortical very fast oscillations (ripples, 80–200 Hz) during seizures: intracellular correlates. *J. Neurophysiol.* 89 841–852 10.1152/jn.00420.2002 12574462

[B38] Hammers A. Asselin M. C. Hinz R. Kitchen I. Brooks D. J. Duncan J. S. (2007). Upregulation of opioid receptor binding following spontaneous epileptic seizures. *Brain* 130 1009–1016 10.1093/brain/awm012 17301080

[B39] Hatanaka N. Tokuno H. Hamada I. Inase M. Ito Y. Imanishi M. (2003). Thalamocortical and intracortical connections of monkey cingulate motor areas. *J. Comp. Neurol.* 462 121–138 10.1002/cne.10720 12761828

[B40] Herz A. Albus K. Metys J. Schubert P. Teschemacher H. (1970). On the central sites for the antinociceptive action of morphine and fentanyl. *Neuropharmacology* 9 539–551 10.1016/0028-3908(70)90004-3 5537123

[B41] Hiller J. M. Pearson J. Simon E. J. (1973). Distribution of stereospecific binding of the potent narcotic analgesic etorphine in the human brain: predominance in the limbic system. *Res. Commun. Chem. Pathol. Pharmacol.* 6 1052–1062 4760880

[B42] Hormuzdi S. G. Pais I. Lebeau F. E. Towers S. K. Rozov A. Buhl E. H. (2001). Impaired electrical signaling disrupts gamma frequency oscillations in connexin 36-deficient mice. *Neuron* 31 487–495 10.1016/S0896-6273(01)00387-7 11516404

[B43] Juhasz C. Nagy F. Watson C. Da Silva E. A. Muzik O. Chugani D. C. (1999). Glucose and [11C] flumazenil positron emission tomography abnormalities of thalamic nuclei in temporal lobe epilepsy. *Neurology* 53 2037–2045 10.1212/WNL.53.9.2037 10599778

[B44] Kahane P. Depaulis A. (2010). Deep brain stimulation in epilepsy: what is next? *Curr. Opin. Neurol.* 23 177–182 10.1097/WCO.0b013e3283374a39 20125010

[B45] Kauffman M. A. Consalvo D. Gonzalez M. D. Kochen S. (2008). Transcriptionally less active prodynorphin promoter alleles are associated with temporal lobe epilepsy: a case–control study and meta-analysis. *Dis. Markers* 24 135–140 10.1155/2008/723723 18334734 PMC3850603

[B46] Kimiskidis V. K. Kugiumtzis D. Papagiannopoulos S. Vlaikidis N. (2013). Transcranial magnetic stimulation (TMS) modulates epileptiform discharges in patients with frontal lobe epilepsy: a preliminary EEG-TMS study. *Int. J. Neural Syst.* 23 1250035 10.1142/S0129065712500359 23273131

[B47] Klaassen A. Glykys J. Maguire J. Labarca C. Mody I. Boulter J. (2006). Seizures and enhanced cortical GABAergic inhibition in two mouse models of human autosomal dominant nocturnal frontal lobe epilepsy. *Proc. Natl. Acad. Sci. U.S.A.* 103 19152–19157 10.1073/pnas.0608215103 17146052 PMC1681351

[B48] Klueva J. Munsch T. Albrecht D. Pape H. C. (2003). Synaptic and non-synaptic mechanisms of amygdala recruitment into temporolimbic epileptiform activities. *Eur. J. Neurosci.* 18 2779–2791 10.1111/j.1460-9568.2003.02984.x 14656327

[B49] Koepp M. J. Richardson M. P. Brooks D. J. Duncan J. S. (1998). Focal cortical release of endogenous opioids during reading-induced seizures. *Lancet* 352 952–955 10.1016/S0140-6736(97)09077-6 9752818

[B50] Kohling R. Gladwell S. J. Bracci E. Vreugdenhil M. Jefferys J. G. (2001). Prolonged epileptiform bursting induced by 0-Mg^(2^^+^^)^ in rat hippocampal slices depends on gap junctional coupling. *Neuroscience* 105 579–587 10.1016/S0306-4522(01)00222-6 11516825

[B51] Kohling R. Vreugdenhil M. Bracci E. Jefferys J. G. (2000). Ictal epileptiform activity is facilitated by hippocampal GABAA receptor-mediated oscillations. *J. Neurosci.* 20 6820–6829 10995826 10.1523/JNEUROSCI.20-18-06820.2000 PMC6772821

[B52] Konopacki J. Kowalczyk T. Golebiewski H. (2004). Electrical coupling underlies theta oscillations recorded in hippocampal formation slices. *Brain Res.* 1019 270–274 10.1016/j.brainres.2004.05.083 15306263

[B53] Kullmann D. M. (2002). The neuronal channelopathies. *Brain* 125 1177–1195 10.1093/brain/awf130 12023309

[B54] Kwan P. Brodie M. J. (2000). Early identification of refractory epilepsy. *N. Engl. J. Med.* 342 314–319 10.1056/NEJM200002033420503 10660394

[B55] Langlois M. Polack P. O. Bernard H. David O. Charpier S. Depaulis A. (2010). Involvement of the thalamic parafascicular nucleus in mesial temporal lobe epilepsy. *J. Neurosci.* 30 16523–16535 10.1523/JNEUROSCI.1109-10.2010 21147992 PMC6634875

[B56] Lee C. M. Chang W. C. Chang K. B. Shyu B. C. (2007). Synaptic organization and input-specific short-term plasticity in anterior cingulate cortical neurons with intact thalamic inputs. *Eur. J. Neurosci.* 25 2847–2861 10.1111/j.1460-9568.2007.05485.x 17561847

[B57] Levin B. Duchowny M. (1991). Childhood obsessive-compulsive disorder and cingulate epilepsy. *Biol. Psychiatry* 30 1049–1055 10.1016/0006-3223(91)90124-5 1756197

[B58] Levitt P. Eagleson K. L. Powell E. M. (2004). Regulation of neocortical interneuron development and the implications for neurodevelopmental disorders. *Trends Neurosci.* 27 400–406 10.1016/j.tins.2004.05.008 15219739

[B59] Lim S. N. Lee S. T. Tsai Y. T. Chen I. A. Tu P. H. Chen J. L. (2007). Electrical stimulation of the anterior nucleus of the thalamus for intractable epilepsy: a long-term follow-up study. *Epilepsia* 48 342–347 10.1111/j.1528-1167.2006.00898.x 17295629

[B60] Loacker S. Sayyah M. Wittmann W. Herzog H. Schwarzer C. (2007). Endogenous dynorphin in epileptogenesis and epilepsy: anticonvulsant net effect via kappa opioid receptors. *Brain* 130 1017–1028 10.1093/brain/awl384 17347252

[B61] Logan J. V. Jacobson G. Sleigh J. W. (eds). (2011). Bridging the Gap – Understanding the Role of Gap Junctions in Seizures. Croatia: InTech

[B62] Lundh H. Nilsson O. Rosen I. (1984). Treatment of Lambert–Eaton syndrome: 3,4-diaminopyridine and pyridostigmine. *Neurology* 34 1324–1330 10.1212/WNL.34.10.1324 6541305

[B63] Madisen L. Mao T. Koch H. Zhuo J. M. Berenyi A. Fujisawa S. (2012). A toolbox of Cre-dependent optogenetic transgenic mice for light-induced activation and silencing. *Nat. Neurosci.* 15 793–802 10.1038/nn.3078 22446880 PMC3337962

[B64] Mann E. O. Mody I. (2008). The multifaceted role of inhibition in epilepsy: seizure-genesis through excessive GABAergic inhibition in autosomal dominant nocturnal frontal lobe epilepsy. *Curr. Opin. Neurol.* 21 155–160 10.1097/WCO.0b013e3282f52f5f 18317273

[B65] Mansour A. Khachaturian H. Lewis M. E. Akil H. Watson S. J. (1987). Autoradiographic differentiation of mu, delta, and kappa opioid receptors in the rat forebrain and midbrain. *J. Neurosci.* 7 2445–2464 3039080 PMC6568954

[B66] Marek B. Kajdaniuk D. Kos-Kudla B. Kapustecki J. Swietochowska E. Ostrowska Z. (2010). Mean daily plasma concentrations of beta-endorphin, leu-enkephalin, ACTH, cortisol, and DHEAS in epileptic patients with complex partial seizures evolving to generalized tonic-clonic seizures. *Endokrynol. Pol.* 61 103–110 20205112

[B67] Marty A. Llano I. (2005). Excitatory effects of GABA in established brain networks. *Trends Neurosci.* 28 284–289 10.1016/j.tins.2005.04.003 15927683

[B68] Mazars G. (1970). Criteria for identifying cingulate epilepsies. *Epilepsia* 11 41–47 10.1111/j.1528-1157.1970.tb03865.x 4987159

[B69] Morrell M. (2006). Brain stimulation for epilepsy: can scheduled or responsive neurostimulation stop seizures? *Curr. Opin. Neurol.* 19 164–168 10.1097/01.wco.0000218233.60217.84 16538091

[B70] Nadkarni S. Devinsky O. (2009). “Cingulate cortex seizures,” in *Cingulate Neurobiology and Disease* ed. Vogt A. (New York: Oxford University Press) 633

[B71] Nagel G. Szellas T. Huhn W. Kateriya S. Adeishvili N. Berthold P. (2003). Channelrhodopsin-2, a directly light-gated cation-selective membrane channel. *Proc. Natl. Acad. Sci. U.S.A.* 100 13940–13945 10.1073/pnas.1936192100 14615590 PMC283525

[B72] Nemani V. M. Binder D. K. (2005). Emerging role of gap junctions in epilepsy. *Histol. Histopathol.* 20 253–259 15578443 10.14670/HH-20.253

[B73] Nitsche M. A. Doemkes S. Karakose T. Antal A. Liebetanz D. Lang N. (2007). Shaping the effects of transcranial direct current stimulation of the human motor cortex. *J. Neurophysiol.* 97 3109–3117 10.1152/jn.01312.2006 17251360

[B74] Nitsche M. A. Paulus W. (2009). Noninvasive brain stimulation protocols in the treatment of epilepsy: current state and perspectives. *Neurotherapeutics* 6 244–250 10.1016/j.nurt.2009.01.003 19332316 PMC5084200

[B75] Nobili L. Francione S. Mai R. Cardinale F. Castana L. Tassi L. (2007). Surgical treatment of drug-resistant nocturnal frontal lobe epilepsy. *Brain* 130 561–573 10.1093/brain/awl322 17124189

[B76] Panuccio G. Antuono M. D. Colosimo A. Cruccu G. Avoli M. (2008a). Different inhibitory modalities shape rhythmic activity generated by anterior cingulate cortex networks. *Biophys. Bioeng. Lett.* 1 3

[B77] Panuccio G. D’Antuono M. Colosimo A. Cruccu G. Avoli M. (2008b). Different inhibitory modalities shape rhythmic activity generated by anterior cingulate cortex networks. *Biophys. Bioeng. Lett.* 1 7

[B78] Panuccio G. Curia G. Colosimo A. Cruccu G. Avoli M. (2009). Epileptiform synchronization in the cingulate cortex. *Epilepsia* 50 521–536 10.1111/j.1528-1167.2008.01779.x 19178556 PMC4879611

[B79] Park E. H. Durand D. M. (2006). Role of potassium lateral diffusion in non-synaptic epilepsy: a computational study. *J. Theor. Biol.* 238 666–682 10.1016/j.jtbi.2005.06.015 16085109

[B80] Perreault P. Avoli M. (1992). 4-aminopyridine-induced epileptiform activity and a GABA-mediated long-lasting depolarization in the rat hippocampus. *J. Neurosci.* 12 104–115 1309571 10.1523/JNEUROSCI.12-01-00104.1992 PMC6575697

[B81] Pert A. Yaksh T. (1974). Sites of morphine induced analgesia in the primate brain: relation to pain pathways. *Brain Res.* 80 135–140 10.1016/0006-8993(74)90731-8 4424093

[B82] Petrovic P. Kalso E. Petersson K. M. Ingvar M. (2002). Placebo and opioid analgesia: imaging a shared neuronal network. *Science* 295 1737–1740 10.1126/science.1067176 11834781

[B83] Pinto D. J. Patrick S. L. Huang W. C. Connors B. W. (2005). Initiation, propagation, and termination of epileptiform activity in rodent neocortex in vitro involve distinct mechanisms. *J. Neurosci.* 25 8131–8140 10.1523/JNEUROSCI.2278-05.2005 16148221 PMC6725540

[B84] Polack P. O. Mahon S. Chavez M. Charpier S. (2009). Inactivation of the somatosensory cortex prevents paroxysmal oscillations in cortical and related thalamic neurons in a genetic model of absence epilepsy. *Cereb. Cortex* 19 2078–2091 10.1093/cercor/bhn237 19276326

[B85] Powell E. M. Campbell D. B. Stanwood G. D. Davis C. Noebels J. L. Levitt P. (2003). Genetic disruption of cortical interneuron development causes region- and GABA cell type-specific deficits, epilepsy, and behavioral dysfunction. *J. Neurosci.* 23 622–631 12533622 10.1523/JNEUROSCI.23-02-00622.2003 PMC6741866

[B86] Prince D. A. (1967). Electrophysiology of “epileptic neurons”. *Electroencephalogr. Clin. Neurophysiol.* 23 83–84 4165587

[B87] Quesney L. F. (1986). Clinical and EEG features of complex partial seizures of temporal lobe origin. *Epilepsia* 27 (Suppl. 2) S27–S45 10.1111/j.1528-1157.1986.tb05738.x 3720711

[B88] Quesney L. F. Constain M. Rasmussen T. Olivier A. Palmini A. (1992). Presurgical EEG investigation in frontal lobe epilepsy. *Epilepsy Res. Suppl.* 5 55–69 1418461

[B89] Racine R. J. (1975). Modification of seizure activity by electrical stimulation: cortical areas. *Electroencephalogr. Clin. Neurophysiol.* 38 1–12 10.1016/0013-4694(75)90204-7 45898

[B90] Rainesalo S. Eriksson K. Saransaari P. Keranen T. (2004). Uptake of GABA and activity of GABA transaminase in blood platelets from children with absence epilepsy. *Neurochem. Res.* 29 1873–1877 10.1023/B:NERE.0000042214.50194.69 15532543

[B91] Romualdi P. Bregola G. Donatini A. Capobianco A. Simonato M. (1999). Region-specific changes in prodynorphin mRNA and ir-dynorphin A levels after kindled seizures. *J. Mol. Neurosci.* 13 69–75 10.1385/JMN:13:1-2:69 10691294

[B92] Ross F. M. Gwyn P. Spanswick D. Davies S. N. (2000). Carbenoxolone depresses spontaneous epileptiform activity in the CA1 region of rat hippocampal slices. *Neuroscience* 100 789–796 10.1016/S0306-4522(00)00346-8 11036212

[B93] Saboory E. Derchansky M. Ismaili M. Jahromi S. S. Brull R. Carlen P. L. (2007). Mechanisms of morphine enhancement of spontaneous seizure activity. *Anesth. Analg.* 105 1729–1735 10.1213/01.ane.0000287675.15225.0b 18042875

[B94] So N. K. (1998). Mesial frontal epilepsy. *Epilepsia* 39(Suppl. 4) S49–S61 10.1111/j.1528-1157.1998.tb05125.x 9637593

[B95] Staley K. J. Proctor W. R. (1999). Modulation of mammalian dendritic GABA(A) receptor function by the kinetics of Cl^-^ and HCO_3_^-^ transport. *J. Physiol.* 519(Pt 3) 693–712 10.1111/j.1469-7793.1999.0693n.x 10457084 PMC2269533

[B96] Steinlein O. K. Mulley J. C. Propping P. Wallace R. H. Phillips H. A. Sutherland G. R. (1995). A missense mutation in the neuronal nicotinic acetylcholine receptor alpha 4 subunit is associated with autosomal dominant nocturnal frontal lobe epilepsy. *Nat. Genet.* 11 201–203 10.1038/ng1095-201 7550350

[B97] Sterman M. B. Shouse M. N. Passouant P. (1982). Sleep and Epilepsy. New York: Academic Press

[B98] Stogmann E. Zimprich A. Baumgartner C. Aull-Watschinger S. Hollt V. Zimprich F. (2002). A functional polymorphism in the prodynorphin gene promotor is associated with temporal lobe epilepsy. *Ann. Neurol.* 51 260–263 10.1002/ana.10108 11835385

[B99] Storm J. F. (1988). Temporal integration by a slowly inactivating K^+^ current in hippocampal neurons. *Nature* 336 379–381 10.1038/336379a0 3194020

[B100] Szabadics J. Varga C. Molnar G. Olah S. Barzo P. Tamas G. (2006). Excitatory effect of GABAergic axo-axonic cells in cortical microcircuits. *Science* 311 233–235 10.1126/science.1121325 16410524

[B101] Szente M. Gajda Z. Said Ali K. Hermesz E. (2002). Involvement of electrical coupling in the in vivo ictal epileptiform activity induced by 4-aminopyridine in the neocortex. *Neuroscience* 115 1067–1078 10.1016/S0306-4522(02)00533-X 12453480

[B102] Tamas G. Buhl E. H. Lorincz A. Somogyi P. (2000). Proximally targeted GABAergic synapses and gap junctions synchronize cortical interneurons. *Nat. Neurosci.* 3 366–371 10.1038/73936 10725926

[B103] Taylor C. P. Dudek F. E. (1982). Synchronous neural afterdischarges in rat hippocampal slices without active chemical synapses. *Science* 218 810–812 10.1126/science.7134978 7134978

[B104] Ulbricht W. Wagner H. H. (1976). Block of potassium channels of the nodal membrane by 4-aminopyridine and its partial removal on depolarization. *Pflugers Arch.* 367 77–87 10.1007/BF00583659 1087404

[B105] Urino T. Hashizume K. Maehara M. Kato K. Okada Y. Hori T. (2010). Epileptic focus stimulation and seizure control in the rat model of kainic acid-induced limbic seizures. *Neurol. Med. Chir. (Tokyo)* 50 355–360 10.2176/nmc.50.355 20505288

[B106] Velasco F. Velasco M. Velasco A. L. Jimenez F. Marquez I. Rise M. (1995). Electrical stimulation of the centromedian thalamic nucleus in control of seizures: long-term studies. *Epilepsia* 36 63–71 10.1111/j.1528-1157.1995.tb01667.x 8001511

[B107] Vogt B. A. (2005). Pain and emotion interactions in subregions of the cingulate gyrus. *Nat. Rev. Neurosci.* 6 533–544 10.1038/nrn1704 15995724 PMC2659949

[B108] Vogt B. A. Pandya D. N. Rosene D. L. (1987). Cingulate cortex of the rhesus monkey: I. Cytoarchitecture and thalamic afferents. *J. Comp. Neurol.* 262 256–270 10.1002/cne.902620207 3624554

[B109] Wada J. A. Hirayasu Y. (2004). Lasting secondary antiepileptogenesis induced by cingulate kindling. *Epilepsia* 45 1308–1316 10.1111/j.0013-9580.2004.19804.x 15509231

[B110] Wada J. A. Tsuchimochi H. (1995). Cingulate kindling in Senegalese baboons, *Papio papio*. *Epilepsia* 36 1142–1151 10.1111/j.1528-1157.1995.tb00474.x 7588460

[B111] Walker J. Storch G. Quach-Wong B. Sonnenfeld J. Aaron G. (2012). Propagation of epileptiform events across the corpus callosum in a cingulate cortical slice preparation. *PLoS ONE* 7:e31415 10.1371/journal.pone.0031415 PMC3283628 22363643

[B112] Wang C. C. Shyu B. C. (2004). Differential projections from the mediodorsal and centrolateral thalamic nuclei to the frontal cortex in rats. *Brain Res.* 995 226–235 10.1016/j.brainres.2003.10.006 14672812

[B113] Williamson P. D. Siegle A. M. Roberts D. W. Thadani V. M. (2000). Neocortical Epilepsies. Philadelphia: Lippincott Williams & Wikins

[B114] Williamson P. D. Spencer D. D. Spencer S. S. Novelly R. A. Mattson R. H. (1985). Complex partial seizures of frontal lobe origin. *Ann. Neurol.* 18 497–504 10.1002/ana.410180413 4073842

[B115] Xiong Z. Q. Stringer J. L. (1999). Astrocytic regulation of the recovery of extracellular potassium after seizures in vivo. *Eur. J. Neurosci.* 11 1677–1684 10.1046/j.1460-9568.1999.00587.x 10215921

[B116] Zaatreh M. M. Spencer D. D. Thompson J. L. Blumenfeld H. Novotny E. J. Mattson R. H. (2002). Frontal lobe tumoral epilepsy: clinical, neurophysiologic features and predictors of surgical outcome. *Epilepsia* 43 727–733 10.1046/j.1528-1157.2002.39501.x 12102675

[B117] Zhang F. Wang L. P. Boyden E. S. Deisseroth K. (2006). Channelrhodopsin-2 and optical control of excitable cells. *Nat. Methods* 3 785–792 10.1038/nmeth936 16990810

[B118] Zubieta J. K. Ketter T. A. Bueller J. A. Xu Y. Kilbourn M. R. Young E. A. (2003). Regulation of human affective responses by anterior cingulate and limbic mu-opioid neurotransmission. *Arch. Gen. Psychiatry* 60 1145–1153 10.1001/archpsyc.60.11.1145 14609890

